# Facebook endodontic groups as potential tools to provide learning opportunities

**DOI:** 10.1002/jdd.13415

**Published:** 2023-11-13

**Authors:** Foujan Pedari, Diego Machado Ardenghi, Renata Grazziotin‐Soares

**Affiliations:** ^1^ Faculty of Dentistry University of British Columbia (UBC) Vancouver British Columbia Canada; ^2^ Division of Restorative Dentistry Department of Oral Health Sciences Faculty of Dentistry University of British Columbia (UBC) Vancouver British Columbia Canada; ^3^ Division of Endodontics Department of Oral Biological and Medical Sciences Faculty of Dentistry University of British Columbia (UBC) Vancouver British Columbia Canada

**Keywords:** dentistry, endodontics, facebook, social media

## 1 PROBLEM

Online media is one of the educational methods that suits well to the Gen Z's preferred learning styles.^1^ Social networking sites are not only predominant in current dental students’ social life, but are interlinked with their self‐learning, search engines, and brainstorming. Albeit several risks exist upon using social media in learning (for instance, dissemination of inaccurate information, violation of privacy, the possibility to ruin one's reputation, and cyberbullying)^2^; Facebook groups were considered tools to provide learning opportunities in higher education.^3^ We hypothesized that this could also be true for dentistry‐endodontics. The problem is that there is a scarcity of information on social media within the dental education literature,^2^ and a paucity of data on content, dynamics, and potential learning opportunities provided by social media endodontic study groups.

## 2 SOLUTION

Aiming to have preliminary information on the potential learning opportunities provided by Facebook endodontic study groups, this approach identified some public/open groups and analyzed their posts (in December 2022). The authors used the first five study groups that appeared on Google after typing “endodontic study groups on Facebook.” The content was publicly available (i.e., content that readers do not need a social networking account or password to access). Upon quantitative (frequency and percentages) and qualitative (authors’ perceptions) measures, this activity assessed *n* = 5 study groups and *n* = 50 posts (the first 10 posts from each group visualized at the day of assessment).

An Excel table aggregated information for each study group (name, creation date, language, number of followers, number of posts in 1 month, type of posts, and main rules), and post (diagnosis, radiographs, clinical pictures, rubber dam use, and final documentation on obturation + restoration).

## 3 RESULTS

Facebook endodontic study groups were created by endodontists (private practice) from Iraq, Egypt, South Africa, and Italy. Four groups were active, and one had its last post in 2019. Groups allowed people who were group members share endodontic cases, articles, or news. The five assessed groups focused on clinical cases. On average, groups had three posts per day, but two of the groups had 10+ posts per day. The biggest group had 198,000 followers. Ethical rules included: “no tolerance for bullying or harassment.” There was no mention if patients had signed a consent form allowing the use of radiographs or pictures in websites.

The posts received few likes (average = 7) and usually no comment; however, the largest group had approximately 100 likes per post with comments. Clinical cases (*n* = 53) were mostly nonsurgical root canal treatment (75.47%) of molars (71.69%). Diagnosis was absent in 67.92%; when present, it was incomplete and/or inaccurate (according to the American Association of Endodontists classification). Sets of radiographs were complete in 60.3% (with bad quality and cropped images). Rubber dam was visualized in 86%. Obturation was 79.24% appropriate (very few small voids and a little of sealer extrusion was considered acceptable/appropriate). Coronal restoration appeared in 81.13%. Find more information in Table 1.

**TABLE 1:** Frequency and percentages of clinical cases (type of treatment and type of tooth), presence of endodontic diagnosis, set of radiographs, presence of rubber dam, quality of root canal obturation, and presence of final restoration that appeared in the 10 assessed posts from each one of the five Facebook endodontic study groups.

*N* = 5 Endodontic study groups on Facebook	Clinical cases *n* = treatment *n* = re‐treatment *n* = type of tooth	Presence of endodontic diagnosis	Complete set of radiographs (pre‐op, working length, cone fit, obturation, and final)	Presence of rubber dam isolation	Quality of root canal obturation	Presence of coronal restoration
Study group 1 (10 posts)	9 treatments	*n* = 3 present	*n* = 4 complete	*n* = 10 present^b^	*n* = 11 appropriate	*n* = 6 present
	3 re‐treatments	*n* = 9 absent	*n* = 8 incomplete	*n* = 0 absent	*n* = 1 inappropriate	*n* = 6 absent
	7 molars					
	2 anterior teeth					
	3 premolars					
Study group 2 (10 posts)	8 treatments	*n* = 7 present	*n* = 9 complete	*n* = 10 present	*n* = 7 appropriate	*n* = 10 present
	2 re‐treatments	*n* = 3 absent	*n* = 1 incomplete	*n* = 0 absent	*n* = 3 inappropriate	*n* = 0 absent
	8 molars					
	1 anterior tooth					
	1 premolar					
Study group 3 (10 posts)	7 treatments	*n* = 3 present	*n* = 6 complete	*n* = 7 present^b^	*n* = 9 appropriate	*n* = 9 present
	4 re‐treatments	*n* = 8 absent	*n* = 5 incomplete	*n* = 3 absent	*n* = 2 inappropriate	*n* = 2 absent
	7 molars					
	1 anterior tooth					
	2 premolars					
	1 unknown^a^					
Study group 4 (10 posts)	8 treatments	*n* = 1 present	*n* = 4 complete	*n* = 7 present	*n* = 6 appropriate	*n* = 9 present
	2 re‐treatments	*n* = 9 absent	*n* = 6 incomplete	*n* = 3 absent	*n* = 4 inappropriate	*n* = 1 absent
	8 molars (including a 3rd)					
	1 premolar					
	1 unknown^a^					
Study group 5 (10 posts)	8 treatments	*n* = 3 present	*n* = 9 complete (1 case had pre‐op CBCT)	*n* = 9 present	*n* = 9 appropriate	*n* = 9 present
	2 re‐treatments	*n* = 7 absent	*n* = 1 incomplete	*n* = 1 absent	*n* = 1 inappropriate	*n* = 1 absent
	8 molars (including a 75)					
	1 anterior (lateral 4 canals)					
	1 unknown^a^					
Total	53 cases	*N* = 17 (32.07%) present	*n* = 32 (60.37%) complete	*N* = 43 (86%) present	*N* = 42 (79.24%) appropriate	*N* = 43 (81.13%) present
	*N* = 40 (75.47%) treatment	*N* = 36 (67.92%) absent	*n* = 21 (39.62%) incomplete	*N* = 7 (14%) absent	*N* = 11 (20.75%) inappropriate	*N* = 10 (18.86%) absent
	*N* = 13 (24.53%) re‐treatment					
	*N* = 38 (71.69%) molars					
	*N* = 5 (9.43%) anterior teeth					
	*N* = 7 (13.20%) premolars					
	*N* = 3 (5.66%) unknown^a^					

^a^ Unknown = authors could not recognize the tooth type/location, as the radiographs were cropped.

^b^ From the 53 posted cases, a total of *N* = 50 rubber dams should appear, because in some cases, adjacent teeth were treated together under the same dam.

Two of the five assessed groups had several followers and engaged individuals in discussions about the clinical cases. Most discussions addressed the techniques, materials, and technologies. In most cases, the radiographic root canal obturation was appropriate, but, contrastingly, patient's medical and dental history, pulpal‐periapical diagnosis, and follow‐up data were missing.

This approach showed that Facebook endodontic groups may provide learning opportunities—in other words, study groups can work as an informal learning environment^4^ and enhance the learning process because dental students may improve their criticism, judgment, and reflexive analysis.^2^, ^5^
